# Phase II trial of SM-88, a cancer metabolism based therapy, in non-metastatic biochemical recurrent prostate cancer

**DOI:** 10.1007/s10637-020-00993-4

**Published:** 2020-09-13

**Authors:** Benjamin A. Gartrell, Mack Roach, Avi Retter, Gerald H. Sokol, Giuseppe Del Priore, Howard I. Scher

**Affiliations:** 1 Albert Einstein College of Medicine, Departments of Oncology and Urology, Montefiore Einstein Center for Cancer Care, Montefiore Medical Center, New York, NY USA; 2 Departments of Radiation Oncology & Urology, University of California San Francisco (UCSF) Helen Diller Family Comprehensive Cancer Center (HDFCC), San Francisco, CA USA; 3 NY Cancer and Blood Specialist, East Setauket, NY USA; 4 Division of Clinical Pharmacology, Uniform Services University of the Health Sciences, Bethesda, MD USA; 5 Florida Cancer Specialist and Research Institute, Fort Myers, FL USA; 6 TYME Inc, New York, NY USA; 7 Genitourinary Oncology Service, Department of Medicine, Memorial Sloan Kettering Cancer Center, New York, NY USA; 8 Department of Medicine, Weill Cornell Medical College, New York, NY USA

**Keywords:** Prostate Cancer, Metabolism based therapy, SM-88

## Abstract

*Background* Androgen deprivation therapy (ADT) is a standard treatment for high-risk biochemically-recurrent, non-metastatic prostate cancer (BRPC) but is not curative and associated with toxicity. Racemetyrosine (SM-88) is an amino-acid analogue used with methoxsalen, phenytoin, and sirolimus (MPS) to enhance SM-88 activity. *Method* A phase 1b/2, open-label trial in BRPC and rising PSA. Patients were given daily SM-88 (230 mg BID), methoxsalen (10 mg), phenytoin (50 mg), and sirolimus (0.5 mg)). Outcome measures included changes in PSA, circulating tumor cells (CTCs) and imaging. *Results* 34 subjects were screened, 23 treated and 21 remained on study for ≥12 weeks. The median PSA was 6.4 ng/ml (range 1.7–80.1); doubling-time 6.2 months (range 1.4–36.6) and baseline testosterone 319.1 ng/ml (range 2.5–913.7). Median duration of therapy was 6.5 months (2.6–14.0). CTCs (median 48.5 cells/4 ml (range 15–268) at baseline) decreased a median of 65.3% in 18 of 19 patients. For patients who achieved an absolute CTC nadir count of <10 cells/4 ml (*n* = 10), disease control was 100% i.e. no metastases or PSA progression, while on trial (*p* = 0.005). PSA fell by ≥50% in 4.3% (1 subject). No patients developed metastatic disease while on treatment (metastases free survival =100%). There were no treatment-related adverse events (AEs) and quality of life was unchanged from baseline on the EORTC QLQ-C30 and QLQ-PR25. Testosterone levels rose slightly on SM-88 and were unrelated to efficacy or toxicity. *Conclusions* Use of SM-88 was associated with disease control while maintaining QOL. SM-88 may delay the need for ADT and the associated hormonal side effects. Larger trials are planned.

Trial registration number, date of registration - NCT02796898, June 13, 2016

## Introduction

Androgen deprivation therapy (ADT) is a standard treatment for a biochemically recurring prostate cancer (BRPC) after definitive local therapy when salvage treatment directed at the prostate or prostate bed is not indicated [1]. ADT uniformly results in declines in prostate-specific antigen (PSA) levels but given alone is not curative. Side effects can be significant and include loss of libido, impotence, hot flashes, fatigue, muscle wasting, bone loss and changes in cognition. Coupled with this toxicity profile, controversy remains as to the optimal timing and duration of ADT to maximize patient benefit. Alternative approaches that control the disease with fewer side effects is a critical unmet need.

Racemetyrosine (SM-88,) is a small molecule amino-acid analogue that has shown antitumor activity in a range of malignancies including metastatic prostate cancer [2]. The anticancer effect is unrelated to testosterone levels in blood or tumor hormone receptor status [3–5]. The use of SM-88 is based on a growing body of literature showing the effects of targeting metabolic pathways that contribute to growth [6–9]. Specificity for cancer cells is high and off target toxicities few [10, 11].

Three agents are used with SM-88: Methoxsalen, Phenytoin, and Sirolimus. These agents are hypothesized to enhance the antineoplastic effects of the drug. Methoxsalen, through several mechanisms including the induction of melanin, is a catalyst for oxidative stress induced cell death [12–15]. Phenytoin is an effective inducer of reactive lipid species [16] which promotes the generation of serum lipids including cholesterol and oxysterols. Sirolimus blocks glucose utilization which results in an increase in amino-acid (SM-88) uptake [17]. None of the added agents have direct antineoplastic effects at the low doses used.

Here we report the safety and potential clinical benefit of SM-88 used with MPS in patients with non-castrate non-metastatic BRPC who were being considered for ADT. Patients were assessed by post-therapy changes in PSA, circulating tumor cells and radiographic imaging.

## Methods

Tyme2016b is a Phase 1b/2, open-label, dose escalation study to evaluate SM-88 (TYME Inc. New York NY) in BRPC opened in June 2016.

Inclusion criteria included males 18 years of age or older with biochemical recurrence after definitive local therapy with curative intent, and no detectable disease on CT and radionuclide bone scan. All patients must have been candidates for ADT based this clinical presentation. Also required was an ECOG score of 1 or less, PSA ≥ 1 ng/mL, and PSA doubling time initially of <9 months or less calculated with a minimum of 3 values obtained 1 or more weeks apart. The doubling time requirement was removed during a subsequent protocol amendment based on investigator input reflecting the observed lack of toxicity. Rising PSA remained an inclusion criteria (https://www.clinicaltrials.gov/ct2/history/NCT02796898). There was no restriction for entry based on serum testosterone levels. However, subjects could not change any hormone related therapies (e.g. ADT etc) prior to entry or add any other cancer treatments while on trial. There was no exclusion based on co-morbid conditions associated with hormone deprivation e.g. hypertension, osteoporosis, etc.

SM-88 was given orally at a dose of 230 mg BID. Subjects also received oral daily doses of MPS at the lowest clinically available doses of repurposed methoxsalen (10 mg), phenytoin (50 mg), and sirolimus (0.5 mg)).

CTC number was determined using an assay with 4 variations based on surface markers and functional assay including an invasion enrichment step followed by multiple parameter flow cytometry [18]. All cells were EpCAM and Dapi +, CD45 –, and phenotypically documented to incorporate invasive matrix gel (Vitatex, LineaRx, Applied DNA, Stony Brook NY). CTCs were sampled on a monthly basis. Data presented is as of the last patient completing 6 months of study therapy in May 2019.

Other endpoints were disease control defined by post-therapy changes in PSA, CTC, and imaging, were assessed by: rising PSA i.e. exclusive of the first 12 weeks of therapy, with >25% increase, and an absolute value >2 ng/ml); and local or metastatic progression by CT and bone scan mandated at study end (6 months after SM-88 start) or earlier if clinically indicated as determined by the local investigator.

Additional outcomes included evaluations of adverse events commonly ascribed to androgen deprivation.

The sample size was based on a single stage design using the exact binomial distribution to allow testing the hypotheses that the approximate overall clinical benefit rate (OCBR) of SM-88 was >25%. The OCBR was defined by PSA level or CTC enumeration, and no imaging worsening after initiation of therapy. For comparison, the assumed spontaneous OCBR was set at 10%. Therefore 33 subjects would yield a type I and type II error rate of <5% and > 80%, respectively.

## Results

**Fig. 1:**
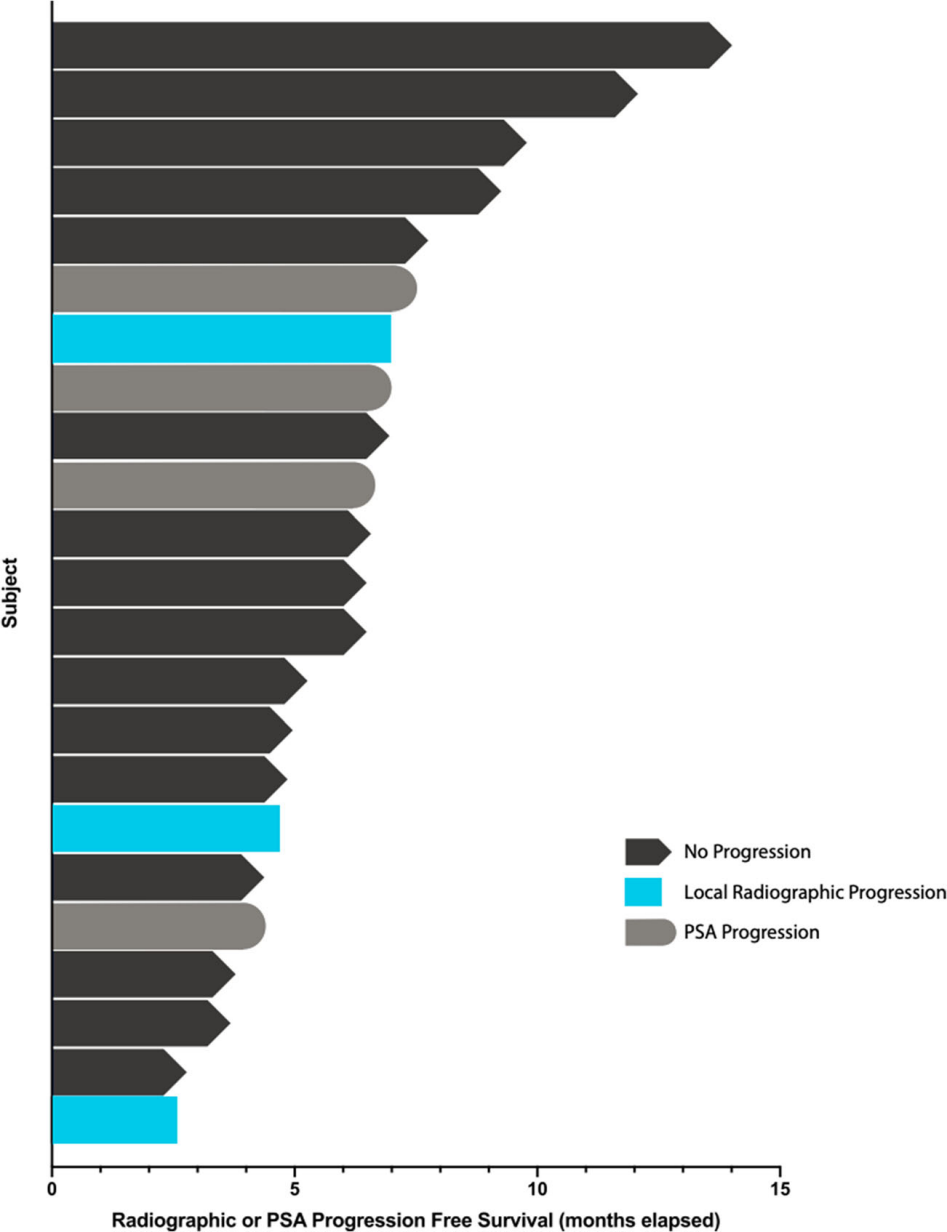
Swimmer’s Plot of Radiographic or PSA Progression Free Survival

As of September 2019, 34 subjects were screened, with 23 subjects enrolled. 21 subjects remained on study for ≥12 weeks. Per protocol, 4 subjects who received the same regimen from Phase 1b portion of the trial were included in the 21 patient overall phase 2 cohort. Table 1 presents the demographic and disease characteristics of the cohort. The population is typical for this disease except for the inclusion of several co-morbid conditions often excluded in clinical trials of hormone based therapy (see Table 2). Median duration of therapy was 6.5 months (range 2.6 to 14.0 months). The cumulative exposure of the entire cohort was >149 months of daily dosing (See Fig. 1). Because of the observed outcomes, the trial was terminated early after clinical experts determined the results justified consideration of moving forward with later stage clinical testing in a randomized trial design.

**Table 1:** Demographics and Baseline Characteristics (*N* = 23)

Age, mean ± SD	70.6 ± 7.4
Weight (kg), mean ± SD	87.4 ± 15.7
BMI, mean ± SD	28.9 ± 4.5
ECOG Performance Status Score, median (range)	0 (0–1)
Race, n (%)	
White	16 (69.6%)
Black	5 (21.7%)
Other	2 (8.7%)
Prior Surgery, n (%)	7 (30.4%)
Prior Radiotherapy, n (%)	14 (60.9%)
Gleason Score, median (range)	7 (6–10)
Gleason Score of 6, n (%)	4 (17.4%)
Gleason Score of 7, n (%)	10 (43.5%)
Gleason Score of 8 to 10, n (%)	7 (30.4%)
Previous Androgen Deprivation Therapy, n (%)	17 (73.9%)
Current Androgen Deprivation Therapy, n (%)	1 (4.3%)
PSA (ng/mL), median (range)	6.4 (1.7–80.1)
PSA Doubling Time (months), median (range)	6.2 (1.4–37.6)
CTCs Detected, n (%)	23 (100%)
Comorbid Disease States, n (%)	
Coronary Artery Disease	3 (13.0%)
Diabetes	4 (17.4%)
Hypertension	15 (65.2%)

**Table 2:** Testosterone related changes

	Baseline	Average on Treatment
Testosterone (ng/ml), mean ± SD	319.1 ± 161.3	359.8 ± 194.5
Weight^a^ (kg), mean ± SD	87.2 ± 15.6	87.6 ± 16.0
Mean Arterial Pressure (mmHg) Normotensive subjects^b^, mean ± SD	90.4 ± 2.6	92.9 ± 6.0
Mean Arterial Pressure (mmHg) Hypertensive subjects^b^, mean ± SD	100.5 ± 5.6	94.2 ± 5.1
QTc (ms), mean ± SD	424.6 ± 25.6	426.4 ± 23.8
Hematocrit (%), mean ± SD	42.6 ± 3.5	41.7 ± 3.5
Glucose (mg/dL), mean ± SD	116.9 ± 56.4	120.0 ± 51.4
Calcium (mg/dL), mean ± SD	9.5 ± 0.4	9.4 ± 0.8
Triglycerides (mg/dL), mean ± SD	104.3 ± 53.9	125.8 ± 41.2
Total Protein (g/dL), mean ± SD	7.0 ± 0.6	6.9 ± 0.4
Albumin (g/dL), mean ± SD	4.4 ± 0.3	4.3 ± 0.2

^a^Last Weight on Treatment was used instead of average of all measures on treatment

^b^Normotensive subjects had both Systolic BP ≤130 and Diastolic BP ≤80; Hypertensive subjects had Systolic BP >130 or Diastolic BP >80

**Fig. 2:**
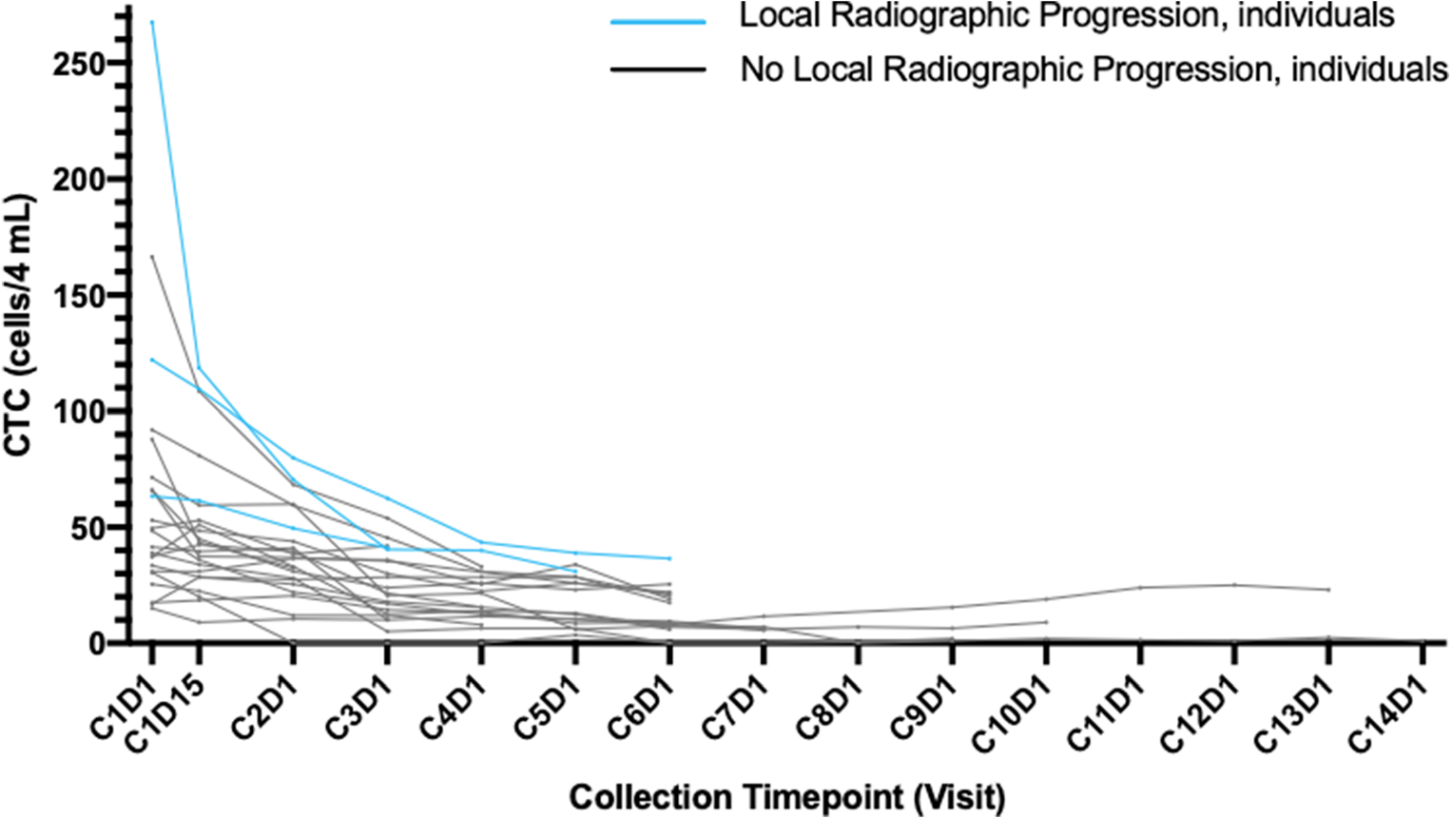
Individual CTC Results By Radiographic Progression

All subjects had PSA values >2 ng/dL at the start of SM-88 treatment on cycle 1, day 1 (range 2.8–80.1); 52% (12/23) of subjects experienced an improvement in PSA doubling time on trial. PSA doubling time improved 34.4% from 6.1 months to 8.2 months for all subjects completing 3 cycles of therapy (*n* = 20). Median baseline PSA for subjects with radiographic progression was 13.4 versus 5.6 for subjects with no radiographic progression (See Figs. 1 and 4). Of all 23 subjects, 19 (82.6%) had some decrease in PSA while on SM-88 between two consecutive cycles, with a median decrease of 7.5% (range 2.7 to 54.8%). One subject (4.3%) had a decrease of 50% or greater in their PSA while on treatment. All patients had detectable CTCs at baseline (See Fig. 2). All patients with available CTC results for at least 3 cycles (*n* = 19) achieved a decrease in CTC from baseline, with a median decrease of 65.3% at the end of 3 cycles, i.e. 12 weeks. One patient did not have a CTC result at 12 weeks but did have results thereafter in subsequent cycles. 94.7% of patients (18/19) maintained CTCs below baseline for the duration of therapy after cycle 3.

Median baseline CTC (per 4 ml) for subjects with radiographic progression (*n* = 3) was 122 cells vs 40 cells/4 ml for subjects with no radiographic progression (*p* < 0.003). Among patients with nadir CTCs >10 cells/4 mL, PSA rose 46.6% on treatment (mean baseline PSA = 15.1 to 22.2 ng/mL at C6D1), while those who achieved nadir CTCs of <10 cells/4 mL had an average PSA rise of only 15.1% (*p* = 0.09).

**Fig. 3:**
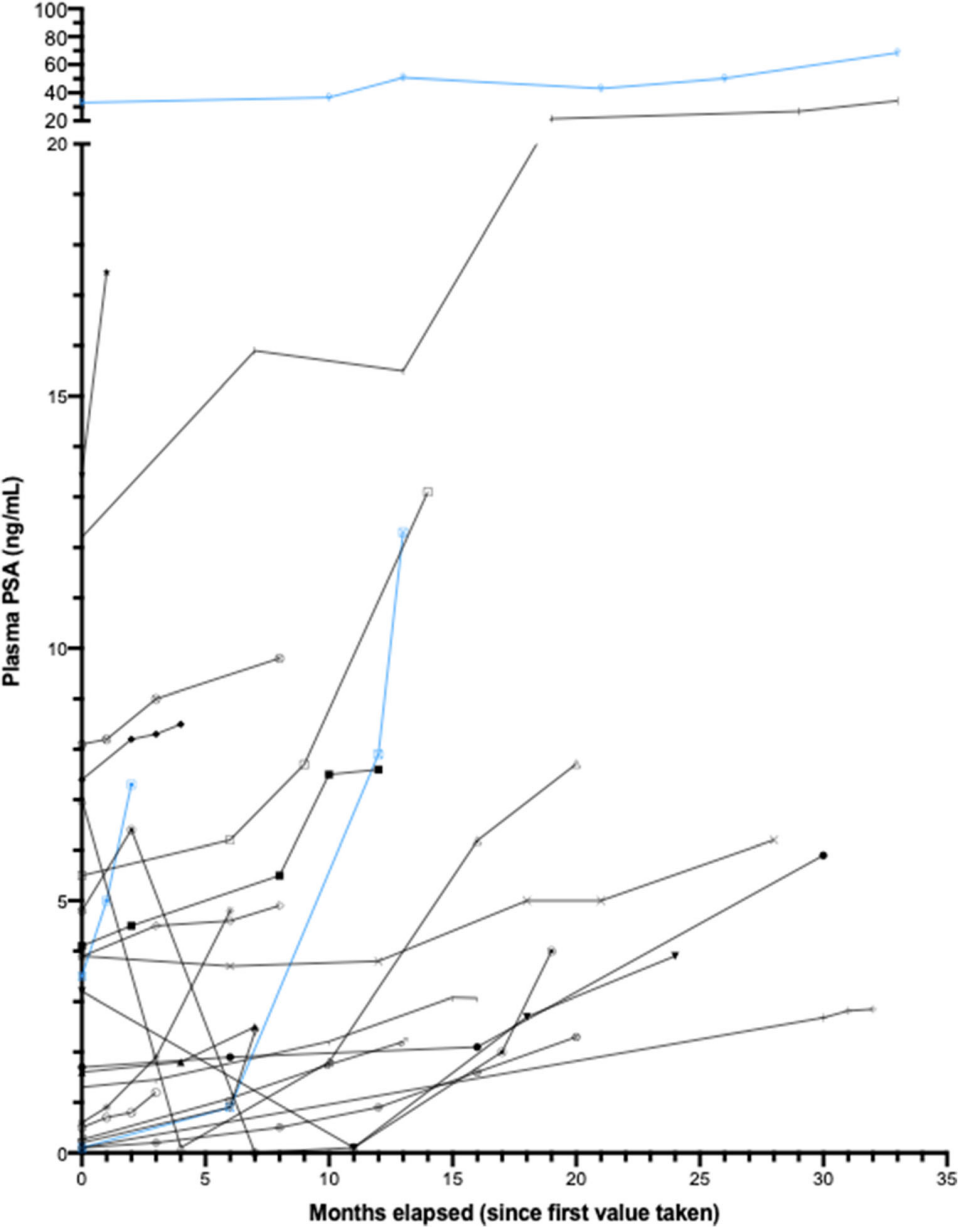
PSA Values Before Treatment

**Fig. 4:**
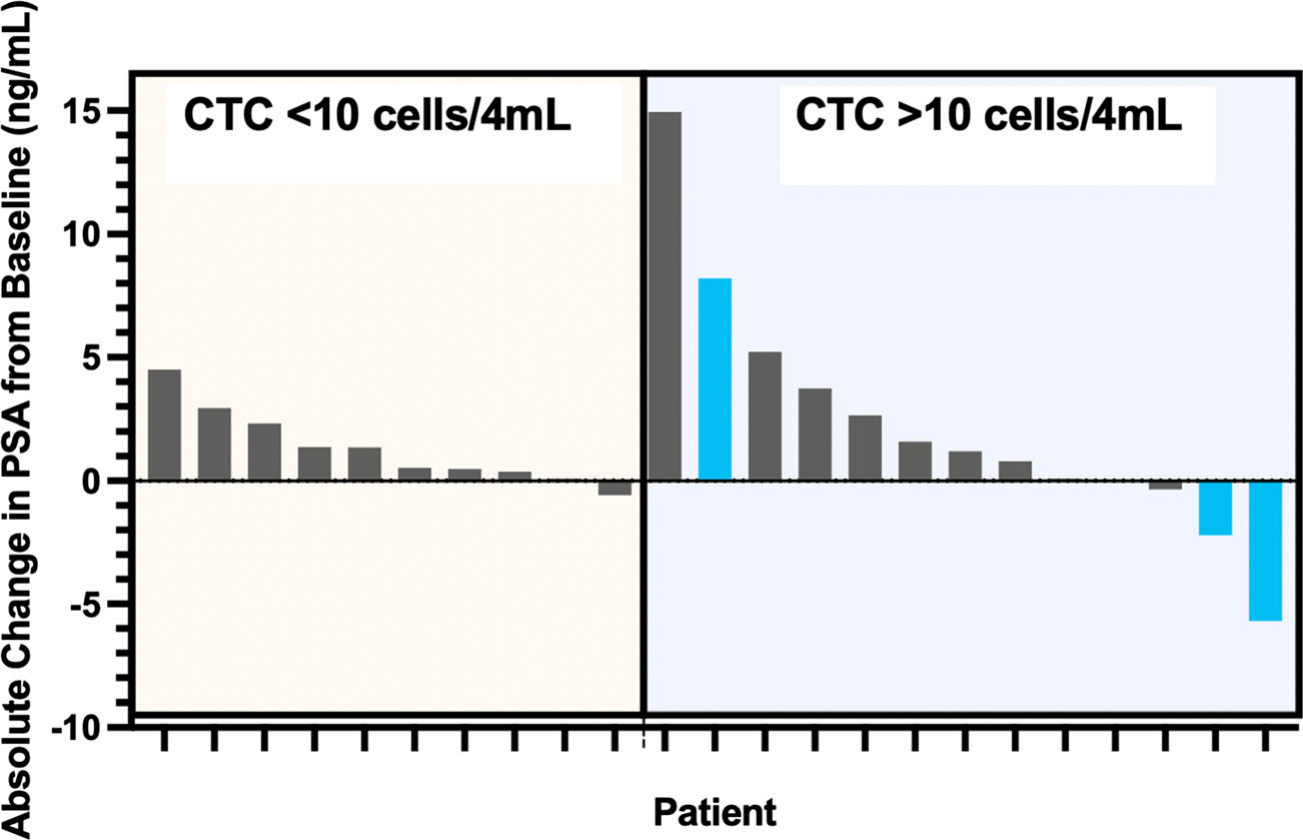
Waterfall Plot of Individual Patient PSA Values – By CTC Group. Blue bars indicate patients with local (one with prostate bed) or regional progression (2 with pelvic node)

Disease control included radiographic PD (all regional *n* = 3) (see Fig. 1) and PSA progression (*n* = 5) (see Fig. 3). For patients who achieved an absolute CTC nadir count of <10cells/4 ml, disease control was 100% (see Fig. 4 waterfall plot of absolute PSA level changes). No subject developed metastatic disease with metastases free survival (MFS) = 100%. All radiographic PD were local (2 pelvic lymph nodes, 1 prostate bed).

Among patients experiencing radiographic progression (n = 3) median baseline urinary N-telopeptide (uNTx) was 72 vs. patients with no radiographic progression (*n* = 20) who had median baseline uNTx of 23.5. uNTx also increased among patients on treatment with nadir CTCs >10 largely due to one of the patients with regional PD. There were no differences in other markers of metastases, including LDH and bone-specific alkaline phosphatase.

Testosterone rose slightly during the trial and was not related to toxicity. Patients without progression (n = 20) had slightly higher testosterone levels at baseline and throughout treatment on SM-88 (median at baseline 343.9, range 2.5 to 624.0 ng/dL; median throughout treatment 351.0 range 2.5 to 913.7 ng/dL, respectively) than those who experienced local radiographic progression (*n* = 3) (median at baseline 295.0, range 2.5 to 442.0 ng/dL; median throughout treatment 319.5, range 2.5 to 433.0 ng/dL).

**Fig. 5:**
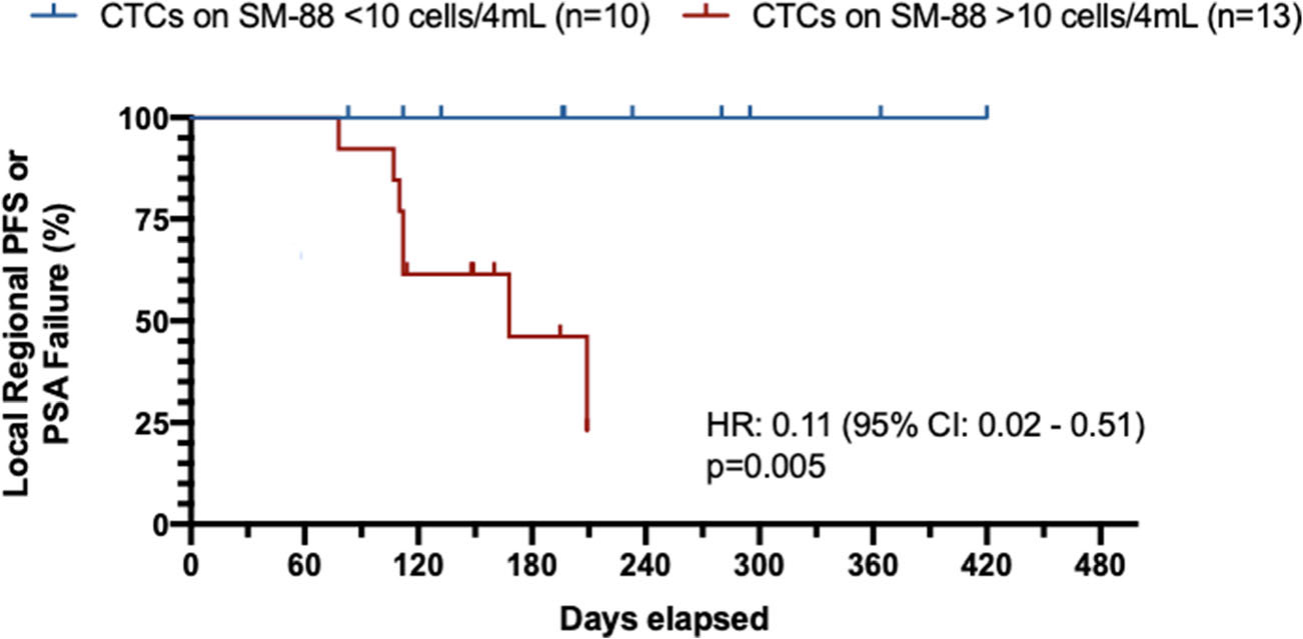
CTC Nadir on SM-88 And PSA or Radiographic Progression

**Fig. 6:**
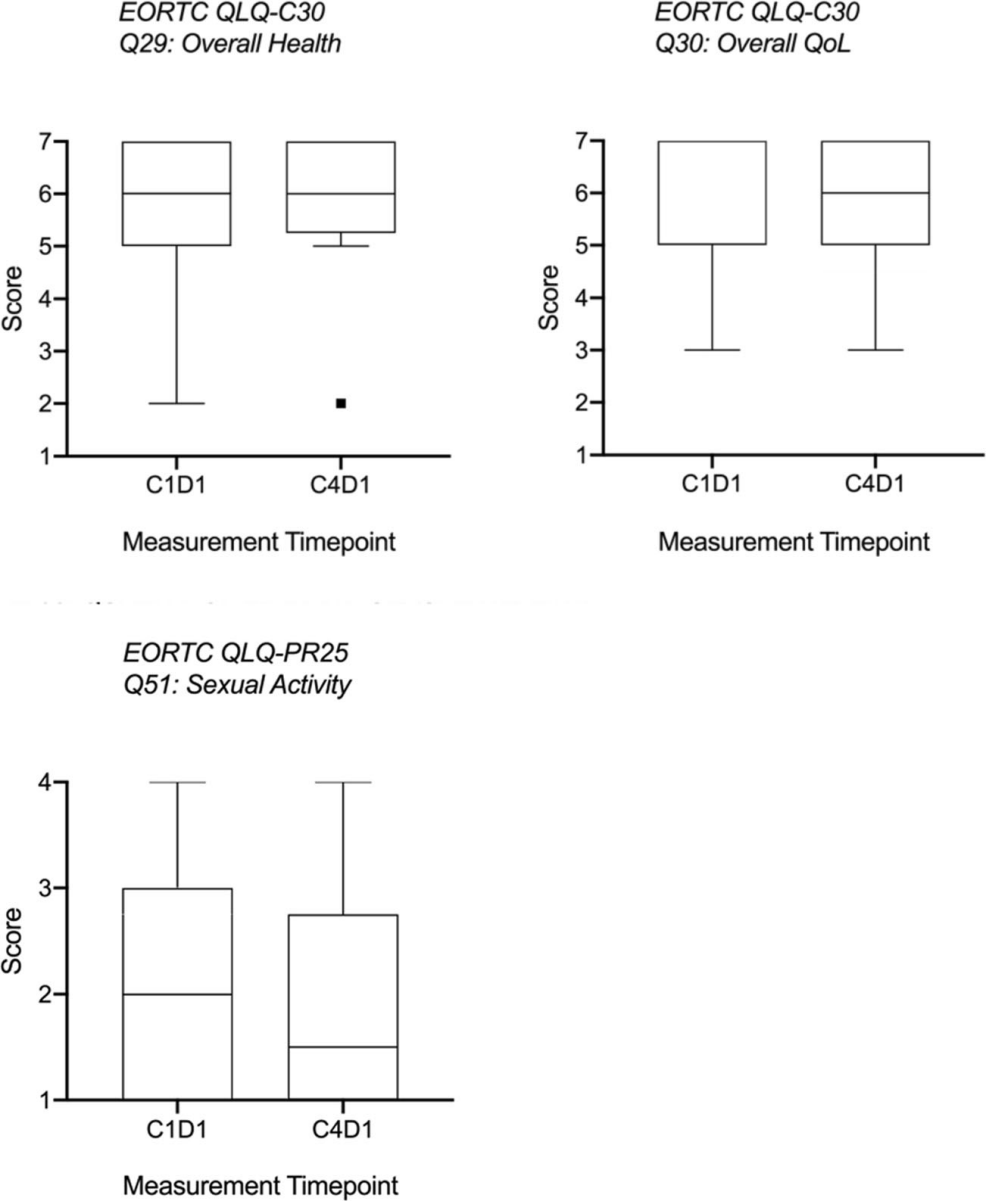
Quality of Life And Sexual Health EORTC Questionnaire

Treatment emergent adverse events were mild (see Tables 2, 3 and 4) and not different from baseline results. There were no grade 4 events and the one grade 3 event (hyperkalemia) was in a patient on diuretics. There was no detectable worsening in any domain of EORTC QLQ-C30 or QLQ-PR25. Globally, overall health and quality of life scores as reported by patients on the EORTC questionnaire administered at every monthly cycle, were relatively high and demonstrated that patients did not experience poor health or low quality of life on SM-88. Generally, patients reported a stable level of sexual activity (see Figs. 5 and 6).

**Table 3:** Treatment emergent adverse events

	Grade 1	Grade 2	Grade 3	Grade 4
Unrelated	10	6	1	0
Possibly/Probably Related	17	1	0	0

**Table 4:** AE Summary

System	AE	Grade	Cause	Rate%
Cardiovascular	Bradycardia	1	Possible	4%
Electrolytes	Hyperkalemia	3	Unrelated	4%
General	Blurred vision	1	Possible	4%
	Chest Pain	1	Unrelated	4%
	Cough, productive	1	Unrelated	4%
	Fatigue (Fatigue, worsening)	1–2	Probable	9%
	Hot flashes	1	Unrelated/Possible	9%
	Lower Jaw/Tooth Swelling and Discomfort	1	Unrelated	4%
	Phimosis	2	Unrelated	4%
	Sore throat	1	Unrelated	4%
	Vitiligo	1	Possible	4%
GI	ALT, elevated	1	Possible	4%
	Bloating, intestinal, intermittent	1	Possible	4%
	Constipation	1	Unrelated	4%
	Diarrhea (Diarrhea, intermittent)	1	Unrelated/Possible	13%
	Diverticulitis	1	Unrelated	4%
	Flatulence	1	Possible	9%
	Nausea	1	Possible/Probable	4%
	Stool, loose (Stools, loose, intermittent)	1	Possible	13%
Hematologic	DVT, bilateral lower extremity	3	Unrelated	4%
	Hematoma, left gluteal muscle	1	Unrelated	4%
Infection/Infestation	Common cold	1	Unrelated	4%
	UTI	2	Unrelated	13%
	Virus, GI	1	Unrelated	4%
Inflammation/Immunologic	Arthritis, gouty	2	Unrelated	4%
	Nodule, prurigo, right arm	1	Unrelated	4%
Metabolic	Lactose intolerance	2	Unrelated	4%
Urinary	Dysuria	1	Unrelated	4%

*AE* adverse event, *ALT* alanine aminotransferase, *DVT* Deep Vein Thrombosis, *GI* gastrointestinal, *UTI* Urinary Tract Infection

## Discussion/conclusions

In this trial, men with previously treated prostate cancer now at risk for metastatic disease based on rising PSA, and who were scheduled to start ADT, were able to delay the start of hormone based therapy while taking an experimental agent, SM-88, [19–21] and thus avoid castration and its consequences.

Since the realization that CTCs may be an important determinant of prostate cancer disease progression and overall survival, agents have been sought to target CTCs without off target effects [22, 23]. Unfortunately, most therapeutic interventions have toxicity associated with hormone deprivation or myelo-suppression [24, 25].

SM-88 is an amino-acid analogue that is thought to leverage cancer’s metabolic dependency on alternative energy sources [26]. Often described as the “Warburg effect”, cancer cells derive most of their energy for metabolism and growth from amino acids and lipids. This observation is the basis for metabolism-based therapies [27].

SM-88 used with MPS as conditioning agents, exploits this phenotype to introduce oxidative stress selectively into cancer cells [2, 7, 8]. This specificity may explain the limited off target effects, and efficacy independent of hormonal deprivation that has now been reported in four cohorts of patients treated with SM-88.

For efficiency, the doses of MPS chosen were the lowest clinically available. These were also the closest to the hypothesized empiric therapeutic levels [17, 28]. These doses are the same or similar to those used in other clinical trials of these repurposed agents. In all cases, the components of MPS are not used for a singular therapeutic objective. To our knowledge, no direct anti-neoplastic effect has been described to any component of MPS.

SM-88 used with MPS is hypothesized to work in a synergistic fashion to drive the death of malignant cells. SM-88 is believed to directly interfere with cancer cells’ ability to synthesize critical proteins [29, 30]. Sirolimus, through inhibition of mTOR, is thought to increase insulin sparing cellular functions, thus forcing cancer cells to meet their metabolic demand by increasing uptake of amino acids and lipids [17, 7, 15, 14]. Phenytoin, through its induction of CYP3A4 can stimulate production of reactive lipid species [16, 31], which may accumulate in the tumor microenvironment, increase the oxidative stress on the tumor, and help drive the cancer towards oxidative related apoptosis. Methoxsalen induces melanin, which is recognized as an electron donor. In the presence of elevated tumor ROS concentrations, melanin may act as a catalyst promoting oxidative stress and facilitating free radical attack [12, 32].

The median duration on therapy was 6.5 months (range 2.6 to 14.0 months) suggesting that a meaningful delay in starting more onerous treatments may be possible on SM-88. Guidance on exactly who may be able to delay subsequent therapies even longer may be indicated by CTCs at baseline, CTC reductions and flattening PSA curves as indicated in this study. CTCs <10cells/4mls was used as an approximation of CTC “zero” based upon direct comparison with older assays indicating that some of the latest methods are 3-20x more sensitive [33–36]. No metastatic disease was detected in this patient population treated with SM-88 despite a median doubling time on entry of 6.2 months. Due to the relatively short period of observation, this finding must be confirmed with longer-term follow-up.

SM-88 may provide disease control without the side effects associated with the ADT standard approach, delaying the need for ADT or other systemic therapies. Unlike with hormone based therapies including ADT, testosterone levels did not have a detectable association with CTC response. For patients with BRPC without metastases, SM-88 could potentially delay the need for more toxic therapies for at least some period of time. Patient-reported overall health, overall quality of life, and sexual activity were largely retained while on treatment.

Alternatively, this compound could be tested in a much earlier disease state, as a means to delay the transition from active surveillance to aggressive treatment (if shown in double-blinded placebo controlled trials to be non-toxic) [37, 38]). Patients at particularly high risk for such a transition (e.g. African American men) might be ideal for such trial [39, 40].

Over the past decade, the “liquid biopsy,” analysis of solid tumor patients has received considerable attention [41]. Among biomarkers, circulating tumor cells have been most intensively analyzed in prostate cancer. The overall goal of CTC-based liquid biopsy testing is to better inform medical decision-making so that patient outcomes are improved. In this trial, comparing the clinical utility of following PSA vs CTCs seems to indicate that CTCs are a more useful parameter in this disease state. Others have reported similar conclusions [34]. Although more research is needed to go beyond its confirmed prognostic utility, this trial is consistent with the hypothesis that CTC reduction is a meaningful intermediate response variable [42].

In addition to reducing CTCs, this trial demonstrated favorable changes in PSA kinetics. This is even more encouraging alongside the observation of rising serum testosterone levels. The men in this trial with BRPC and rising PSA, were generally able to maintain testosterone levels throughout treatment with SM-88. In addition, higher testosterone levels were not associated with worse outcomes. As expected, QOL EORTC questionnaire responses were favorable and consistent with normal testosterone levels.

The majority of adverse events were Grade 1, markedly different from standard ADT. Patient-reported overall health, overall quality of life, and sexual activity were largely retained while on treatment. This is an important distinction from other therapies in two key areas. First, this cohort had no meaningful restriction on co-morbidities. Therefore, these men who enrolled were more representative of a general prostate cancer population than other trials that typically exclude cardiovascular, cerebrovascular, diabetes and other patient selection enhancements [43]. Second, toxicities seen in other trials, whether due to androgen deficiency or cytotoxic chemotherapy, include a wide range of treatment associated severe adverse events ranging from declines in sexual health to death. This unique safety profile may in part be due to the specificity of the theoretical mechanism of action [6].

These results suggest that there may be a clinically meaningful prolongation of the castrate free interval in prostate cancer patients with rising PSA. As an open label phase 2 trial, the results cannot be considered definitive no matter how intriguing. Prospective trials to confirm these results are planned. Potential outcomes will include time to subsequent more toxic therapies and metastases free survival. Based on a high-risk CTC and PSA group, both outcomes are unfortunately all too common and quick to arise in most patients with BRPC. SM-88 has demonstrated possible efficacy and a well-tolerated safety profile that may represent a new option for this patient group seeking a non-cytotoxic, non-hormonal therapy.

## Data Availability

(data transparency) was available to all authors.
